# RNA-seq analysis of an apical meristem time series reveals a critical point in *Arabidopsis thaliana* flower initiation

**DOI:** 10.1186/s12864-015-1688-9

**Published:** 2015-06-18

**Authors:** Anna V. Klepikova, Maria D. Logacheva, Sergey E. Dmitriev, Aleksey A. Penin

**Affiliations:** Institute for Information Transmission Problems of the Russian Academy of Sciences, Moscow, 127051 Russia; A. N. Belozersky Institute of Physico-Chemical Biology, Lomonosov Moscow State University, Moscow, 119991 Russia; Pirogov Russian National Research Medical University, 117997 Moscow, Russia; Engelhardt Institute of Molecular Biology, Russian Academy of Sciences, Moscow, 119991 Russia; Department of Genetics, Faculty of Biology, Lomonosov Moscow State University, Moscow, 119991 Russia

**Keywords:** *Arabidopsis thaliana*, Cell cycle, Flowering, Meristem, Gene expression, RNA-seq

## Abstract

**Background:**

Floral transition is a critical event in the life cycle of a flowering plant as it determines its reproductive success. Despite extensive studies of specific genes that regulate this process, the global changes in transcript expression profiles at the point when a vegetative meristem transitions into an inflorescence have not been reported. We analyzed gene expression during *Arabidopsis thaliana* meristem development under long day conditions from day 7 to 16 after germination in one-day increments.

**Results:**

The dynamics of the expression of the main flowering regulators was consistent with previous reports: notably, the expression of *FLOWERING LOCUS C* (*FLC*) decreased over the course of the time series while expression of *LEAFY* (*LFY*) increased. This analysis revealed a developmental time point between 10 and 12 days after germination where *FLC* expression had decreased but *LFY* expression had not yet increased, which was characterized by a peak in the number of differentially expressed genes. Gene Ontology (GO) enrichment analysis of these genes identified an overrepresentation of genes related to the cell cycle.

**Conclusions:**

We discovered an unprecedented burst of differential expression of cell cycle related genes at one particular point during transition to flowering. We suggest that acceleration of rate of the divisions and partial cell cycling synchronization takes place at this point.

**Electronic supplementary material:**

The online version of this article (doi:10.1186/s12864-015-1688-9) contains supplementary material, which is available to authorized users.

## Background

For annual plants, such as *Arabidopsis thaliana*, proper determination of the flowering time is critical for plant reproductive success because a switch from vegetative to reproductive development is irreversible. Consequently, the transition to flowering is under strict genetic and environmental control [1], with floral initiation being induced by both external (photoperiod- and vernalization-dependent) and internal pathways (autonomous, age- and gibberellin-dependent) [2–4]. Day length has a strong influence on flowering time and for some plants, termed long-day (LD) plants, exceeding of critical day length is necessary for the transition to flowering. In contrast, short-day (SD) plants start to flower only when the day length is shorter than a critical value, and there are also plants that are photoperiod neutral. Dependence of floral induction on day length differs between species and even accessions within a single species [5]. *A. thaliana* is a facultative LD plant, meaning that it proceeds to flowering when day length exceeds a threshold, but it can also flower in a delayed fashion under SD conditions [6, 7].

Analysis of mutants with abnormal flowering time has allowed the identification of genes controlling floral transition [8]. At least 60 genes have been described as participants in flowering regulation [9]. The photoperiod-dependent pathway of floral promotion converges on the gene *CONSTANS* (*CO*), which is known to be expressed in a circadian manner [10]. *CO* is a direct activator of *FLOWERING LOCUS T* (*FT*), a so-called florigen [11, 12]. FT is a small protein that transfers flowering induction signals from leaves into the shoot apical meristem (SAM), where it interacts with the *FLOWERING LOCUS D* (*FD*) product, a bZIP transcription factor, to promote flowering [13, 14]. Another floral integrator is *SUPPRESSOR OF OVEREXPRESSION OF CO 1* (*SOC1*), which is characterized by an early activation in the transition to flowering, marking the switch from a vegetative meristem to inflorescence [15, 16]. The activation of *SOC1* under LD conditions depends on FT and FD [17]. Another element in this complex system is *LEAFY* (*LFY*), a floral integrator whose expression increases in the SAM during the transition to flowering [18, 19]. *LFY* is a positive regulator of *APETALA1 (AP1)* and expression of *AP1* therefore increases later than *LFY* [19]. After flowering initiation, the action of *LFY* and *AP1*, as well as *CAULIFLOWER* (*CAL*) results in the upregulation of genes that control floral organ identity [20–23].

An alternative way to promote flowering involves the exposure of plants to low temperature, a process called vernalization. The key integrator of the vernalization pathway is a MADS-box transcription factor, *FLOWERING LOCUS C* (*FLC*), which functions as a repressor of flowering and whose expression decreases during vernalization [24]. *VERNALIZATION INSENSITIVE 3* (*VIN3*) and *VERNALIZATION 5* (*VRN5*)/*VIN3-like 1* (*VIL1*) genes are known to be involved in chromatin modification that leads to the repression of *FLC* expression [25, 26]. Expression of *FLC* in accessions that are insensitive to cold treatment (such as Columbia or Landsberg *erecta*) is reduced by an autonomous pathway [4, 27, 28]. An interplay between *FLC* and genes involved in photoperiodic activation of flowering has also been reported [29]. Finally, the MADS-box transcription factor *SHORT VEGETATIVE PHASE* (*SVP*) is known to act together with *FLC* to suppress flowering [30].

Despite this extensive knowledge of the behavior of key regulatory genes involved in the transition to flowering, the composition and dynamics of the underlying global genetic networks at the transcriptome level are still poorly understood. A recent study by Torti et al. (2012) focused on an analysis of the *A. thaliana* SAM during transition to flowering and reported gene expression profiles from three developmental stages of meristems, providing useful, although not high-resolution, data. Another study described the development of the inflorescence meristem (IM) and floral meristem, a process that takes place after the transition to flowering [31]; however, the mechanisms involved in transforming a vegetative meristem (VM) into an IM are still unclear.

In this current study we analyzed the dynamics of gene expression in *A. thaliana* meristem during the transition to flowering using RNA sequencing (RNA-seq). This technology allows the determination of genome-wide expression levels as well as the identification of novel transcripts and isoforms. RNA-seq has been successfully used in studies of numerous plant species, including *A. thaliana*, rice (*Oryza sativa*), soybean (*Glycine max*), maize (*Zea mays*) as well as non-model species, such as wild strawberry (*Fragaria vesca*) [32–36]. The most common experimental approach for studies of flowering transition involves growing plants under SD for several days before transferring them to LD. This allows for the synchronization of flowering initiation when plants are placed under permissive photoperiod conditions and thus helps to track processes involved in flowering [29, 37]. It should be noted that under natural growth conditions plants develop without such dramatic increases in day length. We used LD grown plants, which more closely approximates native conditions, and collected meristems at ten developmental stages to obtain a high-resolution data set, thus allowing a detailed evaluation of the processes that accompany the conversion of a VM to an IM.

## Results

### Morphology of the meristem in the course of transition to flowering

**Fig. 1:**
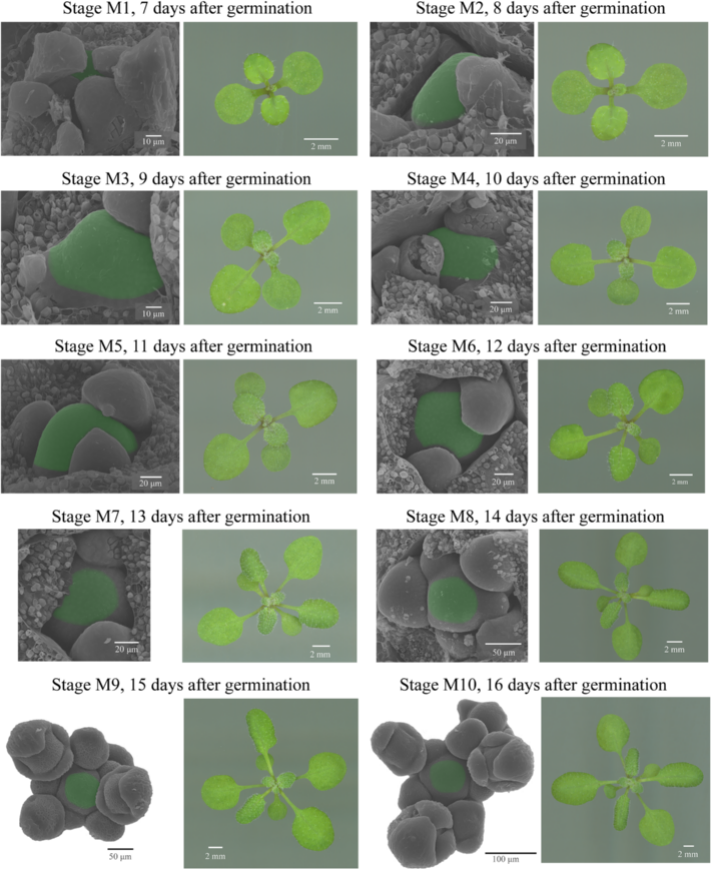
Scanning electron micrographs (SEM) and photographs showing the developmental stages of the shoot apical meristem (SAM). The region that was collected as the meristem sample is false colored green in the SEM images. Leaves covering the meristem were removed. The rosette photographs provide a whole-plant view at the stages of collection

*A. thaliana* SAMs were collected at ten stages from 7 to 16 days after germination (Fig. 1). Due to the developmental variability that occurs even in highly homozygous populations, harvested plants were synchronized by morphological markers: the number and structure of leaves and flowers. Plants at 7 days after germination (referred to as stage M1) had the first and second leaves visible, leaf 3 had trichomes and the last visible leaf primordium was the sixth. Stage M2 (8 days after germination) was characterized by 1-3 visible leaves at the whole-rosette level, the first and second leaves had a central vein with a length 50 % that of the leaf length, leaf 4 had trichomes and the last visible primordium was the eighth. At 9 days after germination (stage M3) leaves 1 and 2 contained a central vein with a length 90 % that of the leaf length, leaf 4 was visible at the whole-rosette level, leaf 6 had trichomes and the last visible primordium was the ninth. At stage M4 (10 days after germination) leaf 5 was visible, leaves 1 and 2 had a central vein with a length 90 % that of the leaf length, leaf 8 had trichomes and the last visible primordium was the thirteenth. Stage M5 (11 days after germination) was characterized by a visible leaf 6 at the whole-rosette level, leaves 1 and 2 were approximately 1 cm in length, leaf 3 had a central vein length that was 50 % that of the total leaf length, leaf 9 had trichomes, and the last visible primordium was the fourteenth. After 12 days of plant development (stage M6) no new organs had emerged, but the whole plant had increased in size. Leaf 3 was 0.7 cm long and had a central vein length 90 % that of the leaf length, leaf 6 was visible at a whole-rosette level, leaf 9 had triсhomes, and the last visible primordium was number 14. The third leaf of plants at 13 days after germination (referred as stage M7) was 1 cm in length, leaf 4 was 0.7 cm, leaf 7 was visible at the whole-rosette level, leaf 10 had triсhomes and the last visible primordium was the sixteenth or seventeenth. Stage M8 (14 days after germination) was characterized by the third leaf measuring 1.5 cm, 1.2 cm leaf 4, leaf 8 was visible, there were triсhomes on the tenth leaf, there were a total of 16 or 17 leaves and 4 floral primordia were present (stage 1 and 2) [38]. At stage M9 (15 days after germination) leaf 9 was visible at the whole-rosette level, leaf 10 had trichomes, there was a total of 16 or 17 leaves and 1–4 floral primordia at stages 2–4 [38]. At stage M10 (16 days after germination) plants had ten visible leaves at the whole-rosette level and 1–4 floral primordia at stages 3–5 [38].

### Transcriptome sequencing of SAMs

**Fig. 2:**
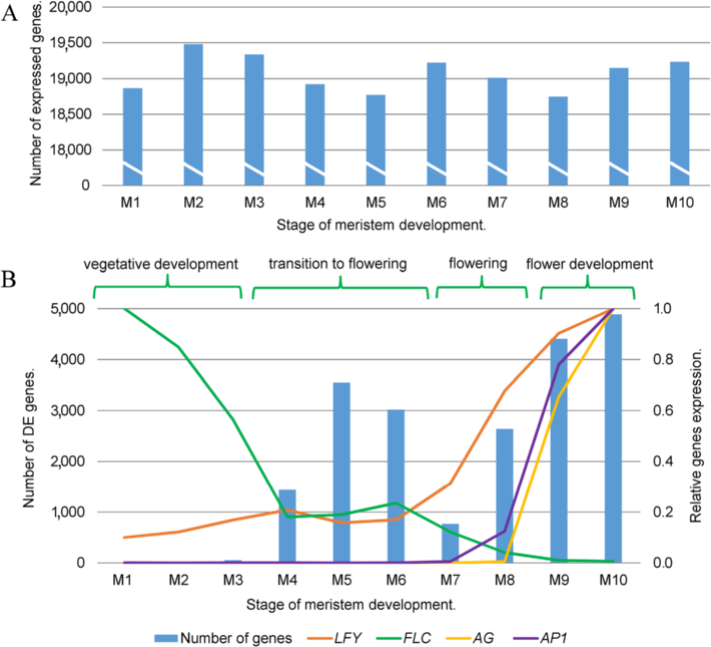
Gene expression during meristem development. (**a**) Number of expressed genes at each stage averaged for two replicates. (**b**) Time series divided in four stages: vegetative development, transition to flowering, flowering and flower development. The number of differentially expressed (DE) genes is indicated for comparison of each stage with the previous stage. Expression profiles of key genes that regulate the flowering process are presented

Pools of samples comprising 15 hand-dissected SAMs for each stage (M1–M10) were harvested in two biological replicates. Total RNA was extracted and used for library construction and sequenced using Illumina protocols. After removal of low-quality reads, >20 million uniquely mapped reads were retained for further analysis from each sample (Additional file 1). Pearson r^2^ correlation values for all replicates varied from 0.96 to 0.99 (Additional file 1), indicating consistency of the raw data. In total, the expression of 21,391 distinct genes was detected, with a slight difference between the numbers of expressed genes between the samples: the highest number of transcripts (19,480) was detected in the stage M2 sample and the lowest (18,750) in the М8 sample (Fig. 2a). A total of 15,312 genes were found to be expressed in all 10 samples (Additional file 1).

### Differential gene expression and transcriptional dynamics of key flowering regulators

The number of differentially expressed (DE) genes varied from 13, between stages M1 and M2, to 4,890 between stages M9 and M10 (Additional file 2). A substantial increase in the number of DE genes (compared with the other pair-wise comparisons) was observed between M4–M5 and M5–M6 (3,552 and 3,019, respectively), while the number when comparing M6 with M7 decreased to 770 (Fig. 2b). A second spike of differential expression was observed from M8 to M10, which may be associated with the formation of floral primordia. To confirm that the data are in agreement with reports in the literature we evaluated the expression dynamics of known key regulators of the transition to flowering and, to this end, the genes for the main negative and positive regulator of flowering (*FLC* and *LFY*, respectively) were chosen as reference markers. According to previously published data, the expression of *FLC* decreases during floral transition while the expression of *LFY* increases [24] and these trends were also observed in our RNA-seq data (Fig. 2b).

The formation of leaf primordia as a part of the vegetative meristem developmental program takes place during stages M1 to M4. At these stages *FLC* is characterized by a five-fold reduction of expression while *LFY* is expressed at low levels. Another negative flowering regulator, *SVP*, is highly expressed in the meristems until the early stages of bolting and in flower primordia [30]. Consistent with this, we found the expression of *SVP* to be high and to increase in this period of the time series. One of the earliest genes activated in the transition to flowering, *SOC1*, was found to show a five-fold increase in expression during stages M1–M4 and a three-fold increase in expression during M3–M4, while *FD* is highly expressed in SAMs before floral induction and undergoes a further increase in expression at later stages [14]. Indeed, we found that between M1 and M4 the expression of *FD* increased 4.5-fold. During the M4–M5 stages, expression of both *FLC* and *LFY* was maintained at low levels, while *SOC1* expression decreased two-fold between M4 and M5, to form a sharp local peak of expression at the M4 stage. Expression of genes from the *SQUAMOSA PROMOTER BINDING LIKE* (*SPL*) family has been shown to increase in SAMs in response to LD conditions [29] and *SPL3*, *SPL4*, *SPL5*, *SPL9* and *SPL15* take part in the transition to flowering upstream of *APETALA1* (*AP1*) [39–42], with *SPL9* and *SPL15* specifically participating in leaf primordium initiation [41]. Accordingly, for these two genes we found a peak of expression at M4 and the onset of a general increase at M6.

From M6 to M8 (where three floral primordia are already formed) *FLC* expression decreased to very low levels and *LFY* expression increased four-fold compared to M6. There was also a continued increase in expression of *SOC1* (1.8-fold). The expression of *SPL4* and *SPL5* started to increase at stage M7, which is consistent with *in situ* hybridization analyses where *SPL3*, *SPL4* and *SPL5* expression was not detected in the vegetative meristem [41]. *SPL9* and *SPL15* expression increased from M6 to M8. In M8 *AP1* expression increased from undetectable levels and *CAL* also expression increased from background levels (Fig. 2b). After the M8 stage an initiation of floral primordia in the SAM occurs. *FLC* expression decreases to almost zero and *LFY* showed a slightly increase in expression. At this point, the activation of genes involved in floral organ identity takes place and we observed that genes such as *APETALA3*, *PISTILLATA* and *AGAMOUS*, all well-known regulators of flower development, showed an increase their transcript abundance from zero to their maximum levels at M10 (Fig. 2b).

Thus, according to the expression dynamics of *FLC*, *LFY* and other regulators of the transition to flowering, our time series can be divided into four main parts: first, stages M1–M4, where *FLC* expression is decreased and *LFY* is expressed at low levels (the stage of vegetative growth of the meristem); second, M4–M6 (a transitional stage, characterized by a parity in *FLC* and *LFY* expression); third, M6–M8 (the stage where the activation of the flowering takes place and when *LFY* expression starts to increase but *FLC* expression is reduced); and fourth, M8-M9 (the stage of flower primordia initiation and development) (Fig. 2b). The transitional stage is of particular interest because of the equal expression levels of negative and positive regulators and a spike in differentially expressed genes in M5. Notably, no new organs formed between the M5 and M6 time points, suggesting that the reprogramming of the meristem from vegetative development to reproductive development is the primary event associated with these patterns. As far as we are aware, this particular developmental stage has not previously been characterized by detailed transcriptome profiling and we propose that a detailed analysis of genes that are up- and downregulated at this point will give new insights into the molecular pathways involved in this transition.

### Gene ontology enrichment analysis of differentially expressed genes at the transitional stage

We characterized the DE genes based on Gene Ontology, several protein domain databases (PIR, InterPro, SMART), the KEGG pathway database as well as other databases with the DAVID Bioinformatics Resources 6.7 [43, 44] (Additional file 2). Enriched terms that characterized the downregulated genes in the M4–M5 comparison and upregulated genes in the M5 and M6 stages, contained categories associated with cytoskeleton organization and movement (microtubule, actin and myosin, kinesin, actin filament-based movement), chromatin modification and DNA replication (helicase, chromatin regulator), ATPase activity (ATP binding, ATPase, AAA+ type, core) and kinases (protein amino acid phosphorylation, serine/threonine protein kinase, active site) (Additional file 2). Enriched categories for the upregulated genes in the M4–M5 comparison and the downregulated genes in the M5–M6 comparison were related to nucleosome assembly (chromatin, histone H4, DNA packaging), tubulins (tubulin complex) and ribosome biogenesis and structure (ribosomal subunit, protein biosynthesis). Genes that were downregulated from stage M4 to M5 belong to categories such as cell cycle regulation and DNA replication (DNA-directed DNA polymerase activity, cell cycle process) while upregulated genes were enriched in the nucleotide metabolism (ATP biosynthetic process, nucleoside triphosphate metabolic process) category.

### Gene clusters

**Fig. 3:**
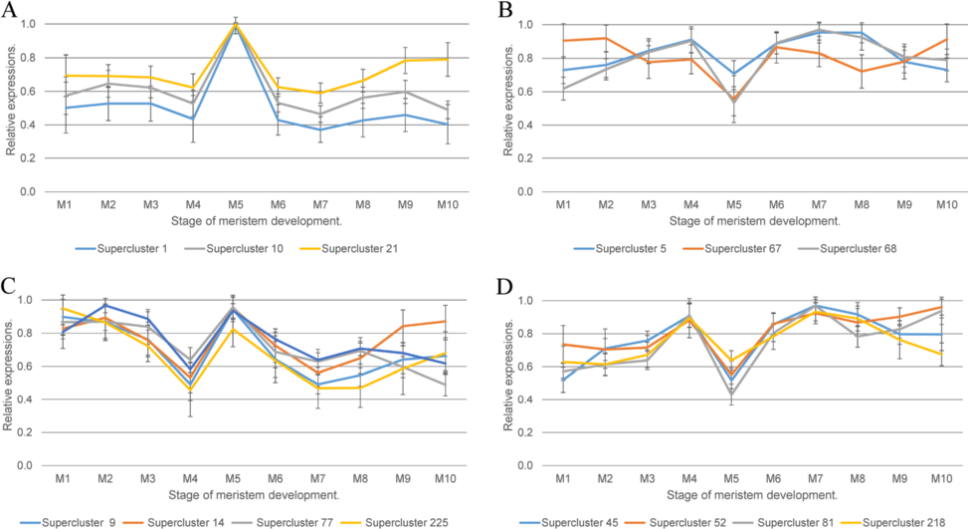
Expression profiles of super-clusters. For specific super-clusters profiles of relative expression values are shown. (**a**) Super-clusters with profiles that have a pronounced spike at M5. (**b**) Superclusters of genes with decreased expression at the M5 stage. (**c**) Profiles with decreased expression at M4 and a subsequent increase at M5. (**d**) Profiles indicating increased expression at M4 and a subsequent decrease at M5. Bars indicate dispersion of normalized gene reads count

To identify groups of genes associated with meristem development, we performed a clustering analysis based on expression profiles and the GO analysis of the resulting data. To obtain the most accurate clustering we used the k-means method with a 1,000 repeats and constructed hierarchical trees based on the distance matrix produced (see Experimental procedures). This allows the separation of genes while avoiding the stochastic nature of k-means clustering. The first clustering step yielded 2,420 clusters containing 18,825 genes (88 % of the expressed genes). The number of genes in the clusters varied from 51 to 5 with a median of 7, which was not sufficient for an effective GO enrichment analysis. Moreover, many clusters had similar expression profiles. Thus, super-clusters were generated using the mean expression profiles of the previously obtained clusters. As a result, 257 super-clusters were generated, containing 16,615 genes (77 % of the expressed genes). For each super-cluster the mean expression profile was calculated and GO enrichment was determined (Additional file 3). Twelve superclusters have expression profiles with generally uniform expression at all stages, with the exception of M5 where expression is distinctly higher and maximal in profile (e.g. superclusters 1, 10, 21, Fig. 3a). Genes in such superclusters are enriched in annotated functions associated with biogenesis of ribosomes, histones, nucleosomes, chromatin modification and mRNA splicing. Superclusters with the opposite profile (i.e. a decrease in expression at stage M5), such as superclusters 5, 67, 68 (Fig. 3b) are enriched in the terms ATPase and nucleotide binding, helicases and pentatricopeptide repeats. Another category comprises superclusters with downregulated genes in M4 and upregulated in M5 (e.g. superclusters 9, 14, 27, 225; Fig. 3c). Terms enriched in these superclusters are associated with biogenesis and structure of ribosomes and photosystems.

Genes whose expression increased in M4 and decreased in M5 fell within superclusters related to the cytoskeleton and cytoskeletal movement (microtubules, kinesins), such as e.g. superclusters 45, 52, 81 and 218 (Fig. 3d).

### Analysis of specific gene classes

The DE gene and super-cluster analysis suggested that genes responsible for chromatin organization, cytoskeletal function, DNA synthesis and cell cycling were upregulated during the initiation of flowering. In order to obtain a more comprehensive view of the dynamics of the gene expression, we chose several groups of genes for a more detailed analysis; specifically those encoding elements of the cytoskeleton (actin, actin-related proteins, actin polymerizing and depolymerizing factors, tubulin, myosin, kinesin and dynein), histone and histone modifiers, DNA and RNA polymerases, cyclins, cyclin-dependent kinases and other genes related to cell-cycle factors. The expression of all actin genes except those that have known functions in floral structures was found to decline at the M4 stage but return to previous levels or increase at M5 (Additional file 4). The actin depolymerizing factors (ADFs) are proteins known to act in the remodeling of actin, thereby controlling its depolymerization [45]. Actin monomers can then be used in new filament formation and ADFs contribute to the dynamic state of the actin network [46]. The *A. thaliana* genome contains 11 ADF genes [47], of which most showed decreased expression at M4 and an increase at M5 (Additional file 4). Only a few ADF genes exhibited dissimilar expression dynamics and these are all known to be expressed in specific plant structures, such as pollen [48].

Villins, of which there are five in *A. thaliana* [49]*,* are actin bundling proteins that can either protect actin from ADFs or promote the severing of actin polymers [50]. In our RNA-seq results all of the villin genes (*VLN1*-*VLN5*) showed a decrease in expression at the M5 stage (Additional file 4). Other gene products involved in stabilizing actin filaments in *A. thaliana* are fimbrins (e.g. FIM1 and FIM2 [51]), which, in the meristems, were found to have a reduced expression from stage M4 to M5 (Additional file 4). Class XI myosins are fast processive molecular motors that play a role in the rapid dynamics of Golgi stacks, mitochondria, peroxisomes and plastids [52–57]. All the myosin genes detected in our study, as well as myosin-like genes, showed a decreased expression at M5 (Additional file 4). While the genes encoding tubulin A and B, proteins that form microtubules, had a peak of expression at the M5 stage, while almost all the kinesins showed a decreased expression at this stage (Additional file 4, Fig. 4). Histones comprised a significantly enriched category in the GO enrichment analysis of genes that increased when comparing M4 and M5 while decreasing in M5–M6 and in super-clusters with a peak in M5. Twenty-eight of the 30 genes encoding histones showed maximal expression at stage M5 (Additional file 5, Fig. 4a and b). Among acetyl transferases, demethylases and methyl transferases, the majority of genes showed a decrease in expression at M5 (Additional file 5), and while genes encoding the subunits of DNA- and RNA-polymerases were downregulated, other non-catalytic subunits common to nuclear DNA-dependent RNA polymerases showed the opposite profile (Additional file 6 and 7). Lastly, we looked at cyclins and cyclin-dependent kinases (CDKs), which function as key regulators of the cell cycle. We determined that cyclins A, B and T had the highest expression at M4 (Additional file 8, Fig. 4b). Taken together, the expression data strongly indicate significant changes in cell cycle progression at the M5 stage.

**Fig. 4:**
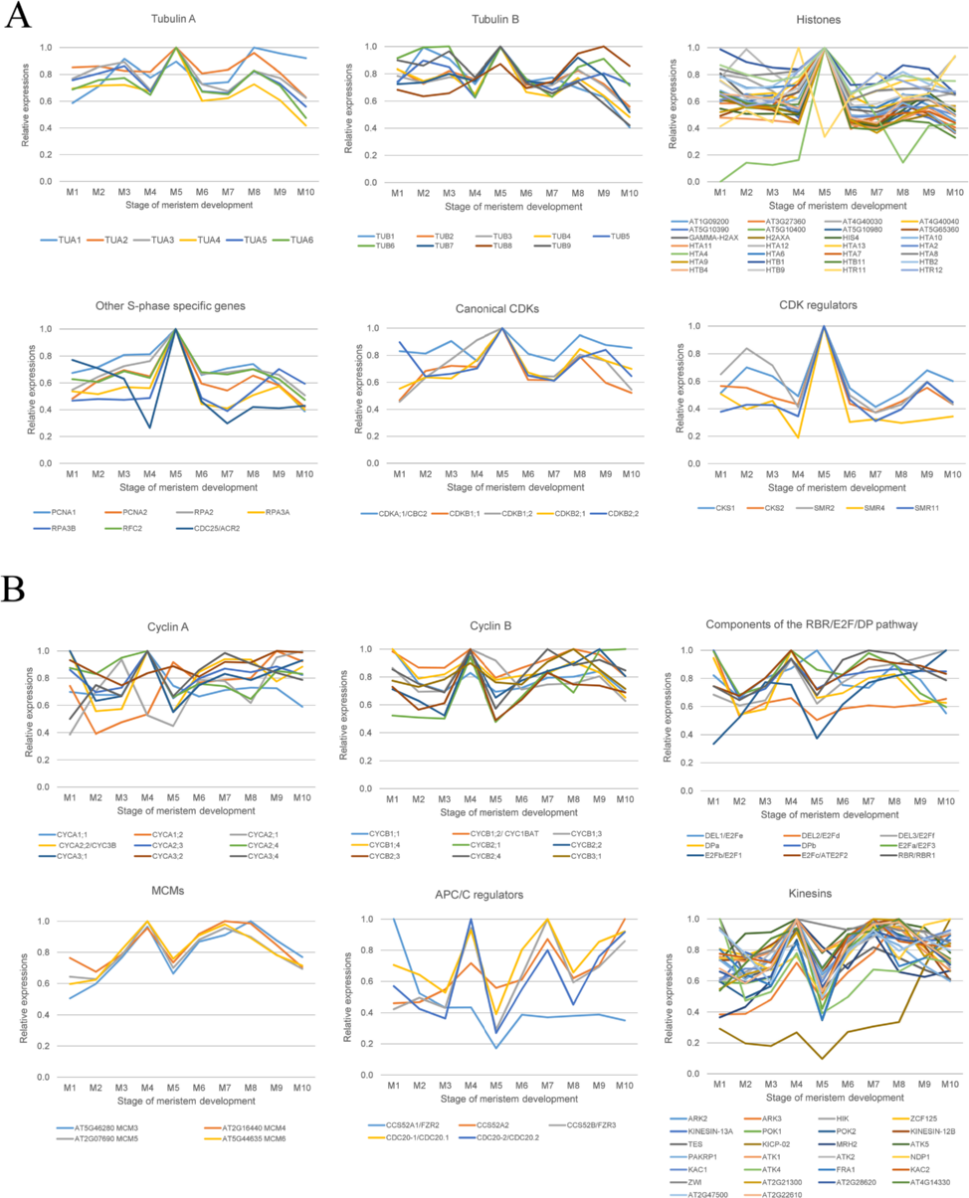
Expression profiles of specific gene classes. Some groups of genes have a synchronized behavior. (**a**) Genes that belong to type (I) of expression pattern have a pronounced peak in M5. (**b**) Genes of type (II) are upregulated in M4 and M6, with a decrease in M5. Relative expression values for the gene classes, normalized to the highest values for each gene, are presented

### Verification of data from the transition stage

Since the RNA-seq data from the initial experiment suggested that the M4–M6 time points are the major stages in the transition to flowering, we repeated the entire experiment for stages M3–M7 (M3N–M7N). Tissues from each stage were collected in two replicates pooled from 15 plants grown under the same conditions and using the same morphological markers as previously described. Normalized total gene read counts and Pearson r^2^ correlation values, which were between 0.93 and 0.99, are shown in Additional file 9. DE genes were identified for each pair of consecutive stages and these matched the lists of DE genes from the first experiment in at least 50 % of the cases. The GO enrichment analysis was similar in all the lists of down- or upregulated genes, with a 100 % match in most cases (Additional file 9). In addition, the super-cluster profiles were very similar between the two experiments, with a similarity median of 0.95 (Additional file 9). For the gene classes described above the similarity median was 0.9 (Additional file 9).

## Discussion

The genes with the most striking changes in their expression profiles included histones, tubulins and cyclins, whose transcription is well known to be highly dependent on the cell cycle stage. Their expression patterns have been well documented in artificially synchronized plant cell suspension cultures [58–60], and so our data point to processes in the development of the SAM involving major cell cycle related events. To our knowledge, such processes have not previously been characterized at the molecular level in the SAM, although changes in the cell proliferation rate occurring at the moment of the transition to flowering have been well documented in cytological studies, as described below.

Two major expression patterns of cell cycle related genes in M4–M6 were: (I) a pronounced peak in M5; and (II) upregulation in M4 followed by a decline in M5 and reversion in M6. Histone gene transcription is well known to be tightly regulated during cell cycle progression ([61] and references therein) and mRNA levels of all histones synchronously increase as cells enter the S phase, but then decrease to lower levels shortly after the end of S phase [60, 62, 63]. Thus, histone mRNA can be used as both a marker of S phase progression and as an indicator of the proliferation index of a given tissue in all organisms [64, 65]. Here, we found that almost all genes encoding histones and uncharacterized histone-like proteins exhibited the type (I) pattern (Fig. 4a, Additional file 5). In addition, genes encoding components of the DNA replication machinery exhibited the group (I) pattern; in plant cell cultures they are known to increase transcript abundance during late G1 and S phases [66]. In our experiment, both *A. thaliana* genes encoding proliferating cell nuclear antigens, PCNAs (*PCNA1* and *PCNA2*) showed a clear single peak at M5 (Fig. 4a, Additional file 6). *RPA2/RPA32A* and two *RPA3*-related genes (*AT3G52630* and *AT4G18590*) encoding subunits of Replication Protein A (a single-stranded DNA binding factor essential for DNA replication) showed a similar expression profile, while genes for variants of the third subunit (RPA70 gene family) did not show differential expression (Fig. 4a, Additional file 6). Other DNA replication related genes, which belonged to group (I), were *RFC2*, encoding replication factor C2 (Fig. 4a, Additional file 6). Expression of the S phase-promoting checkpoint phosphatase *CDC25* homolog (*AT5G03455*) also showed a sharp peak of expression at M5 (Fig. 4a, Additional file 8). This is of particular interest since in plant cell tissue culture this gene has been reported not to show cell cycle regulated expression [60], contradicting studies from other organisms [67]. Interestingly, yeast *CDC25* has been shown to induce flowering in tobacco upon over-expression [68].

Importantly, a pattern similar to those of the above S phase related genes was also observed for transcripts whose presence is usually associated with the late G2 and mitosis stages. For example, the levels of mRNAs encoding α- and β-tubulins clearly peaked at M5, though in this case the profile differed slightly in that the mRNA levels were high not only in M5 but also at early and late stages (M1–M3 and M8–M10, respectively) (Fig. 4a, Additional file 4). During mitosis, higher levels of tubulin monomers are required to form the mitotic spindle, so transcription of tubulin genes is elevated at the late G2/M phase of the cell cycle in many organisms, including plants [58, 62, 63]. This means that in M5 we observe a simultaneous upregulation of S phase and G2/M phase specific genes.

In addition, canonical (A- and B-type) cyclin-dependent kinases also showed type (I) behavior at the M4–M6 stages (Fig. 4a, Additional file 8). Among several classes of CDK genes found in the *A. thaliana* genome, a single *CDKA* gene and four *CDKB* genes are directly involved in cell cycle control [69]. In cell culture, *CDKA;1*, an ortholog of yeast *CDC28* and animal *CDK1* that is exclusively associated with the G1/S-specific cyclins, is constitutively expressed, while plant-specific *CDKB1* and *CDKB2* genes are associated with the G2/M peak of the mitotic cyclins (reviewed in [70]). We found both the *CDKA* and all four *CDKB* genes to be significantly upregulated at the M5 stage. We also observed an elevated expression of two genes encoding scaffold CDK subunits (*CKS1* and *CKS2*) at the same stage (Fig. 4a, Additional file 8). CKSs are components of the CDK-cyclin core complex, which have different expression patterns in cycling plant cells [60, 69]. Finally, three genes for SIAMESE-related cell cycle inhibitors (*SMR2*, *SMR4* and *SMR11*), which are thought to control mitosis, were also substantially upregulated at M5 [69].

**Fig. 5:**
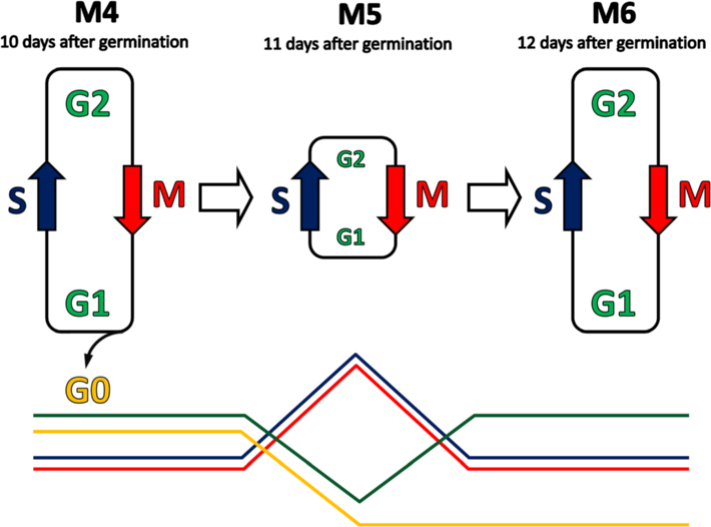
A model explaining the observed changes in the expression level of cell cycle related genes during the floral transition of the shoot apical meristem (SAM). At stages before M4 cells cycle slowly due to long G1 and G2 phases. There is also a large proportion of non-cycling (G0) cells. During floral transition (M4 to M5), the cell cycle duration is shortened at the expense of the G1 and G2 phases, and many G0-arrested cells enter the cell cycle. Subsequently, (after M6), the duration of the cycle increases again. Typical expression patterns of cell cycle regulated genes are shown schematically in the lower panel (those for mitotic genes are red, for S-phase specific genes are dark blue, and G1/G2 and G0 genes are green and yellow, respectively)

These results are congruent with the idea that a burst of cell division occurs in the SAM at the M5 stage, accompanied by a shortening of the G1 and G2 phases (Fig. 5). This was further supported by data from the Gene Ontology Enrichment analysis, which revealed an upregulation of genes associated with nucleotide biosynthesis, ribosome biogenesis, translation, mitochondria and chloroplast components at the same stage (Additional file 2), pointing to an extensive proliferation peaking at this time point [71, 72]. However, the expression profiles of several important cell cycle regulators, including cyclins, appear to complicate this hypothesis. Plants have a complex set of genes encoding cyclins and cyclin-like proteins (Wang et al., 2004; [60, 73] and references therein). In suspension-cultured cells, most A- and B-type (mitotic) cyclins have been shown to have a uniform expression pattern, accumulating in late G2 with a peak in early mitosis [60]. The exceptions are *CYCA3;1*, *CYCA3;2* and *CYCA3;3*, which were predominantly expressed at the G1/S boundary and so actually represent G1 cyclins [60, 74]. The expression of genes encoding for CYCA1s, CYCB1s and CYCB2s are regulated by a common molecular mechanism that involves binding of the R1R2R3-Myb transcription factors MYB3R1 and MYB3R4 to M phase-specific activator (MSA) elements in their promoters [75]. In our time course analysis, at least 13 of these genes, including *CYCA3;1*, showed the type (II) pattern, with a peak of expression at M4 and a subsequent decline at M5 (Fig. 4b, Additional file 8). This correlated well with the expression of a gene encoding the MSA-binding transcription factor MYB3R1, which also showed the type (II) pattern. *CYCA1;2* was the only mitotic cyclin that belonged to group (I), suggesting that this gene has some specific role *in vivo*. The expression of *CYCA3;2* and *CYCB1;3* did not vary significantly, while *CYCA2;1* showed a pattern that was not characterized by either type (I) or (II). Thus, most of the Cyclin A and Cyclin B genes belonged to the type (II) group.

Genes encoding D-type cyclins did not show such a uniform expression profile. In synchronized suspension cell culture, they have been shown to have peaks of expression during the G1 phase and G1/S transition, although with a diversity of profiles: *CYCD5;1* and *CYCD3;3* mRNAs accumulate in early G1 and levels then decline as cells move towards the S phase, whereas *CYCD4;1*, *CYCD4;2* and *CYCD3;1* mRNAs accumulate in late G1 and peak at the G1/S boundary [60, 66]. Of the 9 cyclins in this class for which we were able to detect expression, two (*CYCD3;2* and *CYCD3;3*) exhibited type (I) expression, three (*CYCD2;1*, *CYCD4;2* and *CYCD6;1*) demonstrated high expression only in the M1 stage, one (*CYCD1;1*) peaked at M9–M10, and the others showed no significant changes throughout the time period investigated (Additional file 8).

Most of the genes encoding E2F/DP/RB pathway components, which are known to be G1/S phase regulated (DPa, DPb, E2Fa, E2Fb, E2Fc, RBR, WEE1 kinase and DEL3, see [60]), also belonged to the type (II) expression category (Fig. 4b, Additional file 8). All three genes encoding APC activators for which we were able to detect transcripts (*CDC20-1*, *CDC20-2* and *CCS52B*) were similarly type (II), even though they demonstrated a M phase specific expression in cell culture [60].

Even more intriguing was the observation that some genes that were expressed at the same stage during cell cycle progression in plant cell culture showed the contrasting pattern of expression during the M4–M5 stage in our study of expression in SAMs. For example, tubulins and kinesins have previously been reported to show a simultaneous peak of expression in the G2/M phase of the cell cycle [58, 60, 66]. However, in our study, tubulin genes exhibited type (I) expression, while all genes encoding kinesins clearly demonstrated type (II) expression (Fig. 4a and b, Additional file 4). The same was true for the PCNA and MCM families, both of which comprise S phase specific genes [66]. While *PCNA1* and *PCNA2* grouped with the type (I) genes (see above), several MCM genes (*MCM3*, *MCM4*, *MCM5*, *MCM6*) had the type (II) expression pattern (Fig. 4a and b, Additional file 6). We suggest that the antitropic expression of genes encoding important cell cycle regulators occurring in the M4 stage can be explained by major developmental and physiological reorganization within the cells, which at that point are preparing to enter into extensive proliferation that occurs at M5. We propose that at the M4 stage a fraction of cells from the vegetative meristem, which are slowly cycling and residing in the G2 phase, are activated and begin to move synchronously toward mitosis, while those cells residing in G1 start to prepare to enter the S phase. In M5, they rapidly cycle without long G1 or G2 phases, while in M6 and subsequent stages the proliferation rate returns to the normal level. We suggest that ‘reformatting’ of the cell cycle in M4 SAMs underlies the non-canonical pattern of gene expression at this stage. At M5, some non-dividing (G0) cells that were present in the SAM may also have entered into the division phase, as suggested by expression of some D-type cyclins.

The dramatic changes in the cell proliferation status of cells in the vegetative SAM during the floral transition has previously been reported, but has not yet been characterized in molecular terms. Specifically, since the 1960s, several groups have described this phenomenon extensively using cytological approaches, mostly based on studies of *Silene coeli-rosa* (by R. Lyndon and D. Francis groups) and *Sinapis alba* L. (by G. Bernier group), but also in *A. thaliana* and other species ([76–79]. In all these studies, the floral transition was induced by manipulating the photoperiod and as a result of these analyses the authors drew several important conclusions. First, the rate of cell division in the SAM sharply increased during the transition to flowering, with cell doubling time decreasing from 20–80 h (depending on species and the SAM zone) to only a few hours (see [78–82] and references therein). This dramatic reduction in the cell cycle duration was achieved mostly by a shortening of the G1 and G2 phases [78, 81, 83], although some authors also reported a shortening of the S phase [78, 84]. The latter was essentially achieved by an activation of latent DNA replication origins (i.e. an increase of the number of replicon origins per unit length of DNA) and was observed immediately prior to commitment to flowering [84, 85]. In contrast, the changes in the duration of G1 and G2 proceeded in several stages. G2 was the longest phase of the cell cycle in the vegetative SAM and G1 was the longest at later stages [86]. According to one detailed study, at the first stage a reduction in the length of the G2 phase of the rapidly cycling cells occurred together with a return of non-cycling G2 cells to the division cycle [78]. This contributed to the first mitotic wave in the inflorescence meristem. Subsequently, a shortening of the G1 phase of the rapidly cycling cells and the release of some non-cycling G0/G1 cells into the S phase occurred contributing to the second mitotic wave. These observations are consistent with our hypothesis. Second, the changes in the evoked meristem observed at the cytological level included a partial synchronization of cell divisions in the SAM (see [83, 86], and references therein). This synchronization however could be suppressed under certain conditions [87] indicating that it may be a side effect of the activation of cell division. The third observation was that the cell population of the meristem was heterogeneous, including zones of rapidly cycling and non-cycling cells. During the floral transition, the cell proliferation rate changed to differing extents between these zones [80, 81, 86]. All these processes contribute to the complex picture we observed at the molecular level.

We note that since all the above observations were made in an SD/LD-induced system, it was not possible to accurately determine whether the changes in cell division were caused by the induction stimulus itself, or indeed reflected authentic processes related to floral transition. In contrast, in the current study, with our morphology-driven synchronization approach we were able to determine unambiguously that the cell cycle related events are an integral part of the flowering program.

## Conclusions

This study provides a comprehensive high-resolution characterization of gene expression during floral transition in *Arabidopsis thaliana* meristem. We found dramatic increase in number of differentially expressed genes at the point when a vegetative meristem transitions into an inflorescence. Using analysis based on clustering of expression profiles we found coordinated changes in expression of genes involved in cell cycle. We hypothesize that at this point a subset of the meristematic cells experiences a forced exit from G0 and/or G1 and G2 shortening at day 10 and then an acceleration of the cell cycle occurs at day 11, which may be linked to meristem reorganization preceding activation of *LFY*. We expect that further experiments will validate and elucidate the mechanism underlying these events.

## Methods

### Plant growth and sample preparation

A single *A. thaliana* plant (accession CS70000; Col-0) was grown in conditions that prevented outcrossing. One seed from a self-pollinated flower was selected and the plant was grown to maturity, and this procedure was repeated three times to increase the homozygosity. To promote germination, seeds were stratified on 1/2 vermiculite:soil at 4 °C for 5 days. For SAM collection, plants were grown in a climate chamber (POL-EKO Aparatura, Poland) under a 16-h light/8-h dark cycle at 22 °C and 50 % relative humidity, using Philips Master TL5 HO 54 W/840 lamps as the light source and a 27 cm distance from the lamps to the plants. To obtain synchronized plants at different developmental stages, plants were harvested using morphological markers for 7–16 day old plants. Hand-dissected SAMs were fixed in RNAlater (Qiagen, Germany) in two biological replicates with tissue from 15 individuals in each sample. Collection of material was conducted from 10 to 11 h after dawn (Zeitgeber time (ZT) 10–11). Each meristem was placed in RNAlater no more than 1.5 min after harvesting. The stages from 9–14 day old plants were collected in two replicates for the second independent experiment under the same conditions.

### RNA extraction and sequencing

Total RNA extraction was performed using an RNeasy Plant Kit (Qiagen, Germany) following the manufacturer’s protocol. Illumina cDNA libraries were constructed with the TruSeq RNA Sample Prep Kits v2 (Illumina, USA) following the manufacturer’s protocol in 0.4 of the recommended volume due to the small amounts of RNA in the samples. Sequencing of the cDNA libraries was performed using an Illumina HiSeq2000 with a 50 bp read length and a sequence depth of 20 million uniquely mapped reads for the first experiment and 15 million for the second experiment.

### Sequence trimming, mapping and expression level determination

Reads were trimmed using the CLC Genomics Workbench 6.5.1 with the parameters “quality scores - 0.005; trim ambiguous nucleotides – 2; remove 5’ terminal nucleotides – 1; remove 3’ terminal nucleotides – 1; discard reads below length 25”. Trimmed reads were mapped using the RNA-seq mapping algorithm implemented in CLC Genomics Workbench to the reference *A. thaliana* genome (TAIR10) allowing only unique mapping with a maximum of two mismatches. For each gene, total gene reads (TGR) was determined as the sum of all reads mapped on this gene. To avoid bias due to different library sizes, TGR values were normalized by size factor as described in Anders and Huber, 2010 [88].

### Identification of differentially expressed genes

Differentially expressed (DE) genes were identified for each pair of consecutive stages using the R package “DESeq” [88]. A false discovery rate (FDR) of 0.05 was chosen as the threshold for DE gene detection.

### Quantitative PCR

For verification of RNA-seq results, we performed qRT-PCR analysis of the expression of four genes (*LFY, FLC, AP1, AG*) that are markers of meristem development stages. *AT4G33380* and *AT4G34270*, two genes that were found to be stable under wide range of tissues, developmental stages and conditions [89], were taken as reference. RNA was extracted as described above, cDNA was synthesized using SuperScript II (Invitrogen). PCR was performed using 2x KAPA SYBR FAST qPCR Master Mix (Kapa Biosystems, South Africa) on Eco Real-Time PCR (Illumina, USA) under following program: 95 °C – 5 min (1 cycle), 95 °C – 10 s, 60 °C – 30 s (45 cycles). Gene expression levels were calculated using ddCt method [90]. Primer sequences, as well as detailed results, are listed in the Additional file 10.

### Gene ontology enrichment analysis

For each DE gene list, downregulated and upregulated genes were annotated using Gene Ontology (GO) enrichment analysis. An enrichment analysis was also performed using key words and protein domain were identified with the DAVID gene functional annotation tool with an FDR value of 0.05 as the threshold of significance [43, 44].

### Relative expression values

Normalization using the maximum value from the gene clustering and expression profiles was performed. The average for replicates normalized to TGR counts was divided by the maximum value of counts for each gene. For construction of expression profiles from the second experiment, which was compared to the profiles from the first experiment, normalized TGR counts for samples M3N-M7N were normalized by the maximum value together with samples M1, M2 and M8–M10.

### Clustering

Genes that were expressed in both replicates of at least one sample at 5 or more normalized TGR counts were selected for further analyses. These genes were clustered using k-means, with k = 1500 and 1,000 repeats of the “k-means” function using the R software package (package “stats”) [91]. A table with 1,000 numbers of clusters for each gene was produced and used to construct a matrix of distances. For each pair of genes the term N was used to designate the number of times these genes occurred in the same cluster and 1,000-N was used as the measure of distances between genes. The distance matrix was treated with the R function “as.dist” and a hierarchical tree was constructed using the function “hclust” from the R package “fastcluster” [92]. The resulting tree was cut with the “cutreeDynamic” function from the R package “dynamicTreeCut” and minimum cluster size was 5 [93]. Due to the small number of genes in each cluster, a GO enrichment analysis was not effective. There were also many clusters with a similar expression profile that could be combined, so to reduce the number of clusters a mean cluster profile was calculated for each cluster as a mean of the TGR count of all the genes in a cluster for each stage. Mean cluster profiles were clustered by k-means with k = 100, and a matrix of distances between cluster profiles was defined with the same approach as for the genes. A hierarchical tree was calculated based on this distance matrix and cut with a minimum cluster size of 5. For the resulting super-clusters, the mean super-cluster profile was counted as similar to the mean TGR count of all genes in a super-cluster at each stage.

To determine the overlap of gene or super-cluster expression profiles from the first and second experiment, the measure of the distance for single profiles was defined as the mean squared distance between each expression value in the first and second experiment, thereby giving as a measure of similarity a “1-measure of distance” value.

### Scanning electron microscopy (SEM)

After fixation in 70 % ethanol, plants were transferred to 80 % ethanol for 15 min, 96 % ethanol for 15 min, ethanol:acetone (1:1) for 1 h and then acetone 3 times for 30 min. Imaging was carried out using two electron microscopes, CamScan 4S (CamScan, Cambridge, UK) and JSM-6380 (JEOL, Tokyo, Japan), with an acceleration voltage of 15–20 kV. SEM images were treated and colored using Adobe Photoshop.

### Availability of supporting data

The Illumina sequence reads have been deposited into NCBI Sequence Read Archive [project ID PRJNA268115].

## Additional files

Additional file 1:
**Statistics of RNA-seq, normalized total gene read counts, correlations between samples and expressed genes lists.**
Additional file 2:
**Statistics of differentially expressed genes detection, list of DE genes and Gene Ontology enrichment for each comparison.**
Additional file 3:
**List of genes, mean expression profile and Gene Ontology enrichment for superclusters.**
Additional file 4:
**Lists of genes and their expression profiles for genes related to cytoskeleton.**
Additional file 5:
**Lists of genes and their expression profiles for genes related to histones.**
Additional file 6:
**Lists of genes and their expression profiles for genes related to DNA polymerases.**
Additional file 7:
**Lists of genes and their expression profiles for genes related to RNA polymerases.**
Additional file 8:
**Lists of genes and their expression profiles for genes related to cell cycle.**
Additional file 9:
**Statistics of RNA-seq, normalized total gene read counts, correlations between samples, list of DE genes and Gene Ontology enrichment for comparison between each sample, mean expression profile for superclusters and expression profiles of gene groups for second experiment.**
Additional file 10:
**Description of qRT-PCR results and technical details of the assay.**

## Abbreviations

GO: Gene Ontology
SD: Short Day
LD: Long Day
SAM: Shoot Apical Meristem
IM: Inflorescence meristem
VM: Vegetative meristem
RNA-seq: RNA sequencing
DE: Differentially expressed
ADF: Actin depolymerizing factor
CDK: Cyclin-dependent kinase
